# Legal Aspects of Migraine in the Workplace

**DOI:** 10.1007/s11916-022-01095-x

**Published:** 2022-11-18

**Authors:** Nina Riggins, Lorenzo Paris

**Affiliations:** 1 UCSD Headache Center, University of California, San Diego, USA; 2 UCSD Medical School, San Diego, CA USA; 3 California, State Bar, San Francisco, USA

**Keywords:** Headache, Migraine, Workplace, Paid sick leave, Short term disability insurance, Long term disability insurance, Americans with Disability Act (ADA), Family and Medical Leave Act (FMLA), Social Security Disability (SSDI), Supplemental Security Income (SSI), Workers’ compensation, Legal rights of persons living with migraine, Employers, Employees, Law

## Abstract

**Purpose of Review:**

This study explores legal aspects of migraine in the workplace. While the high prevalence of migraine is well-documented, its effects on the livelihoods of those living with the disease are less understood. Because migraine symptoms are often invisible, discussions concerning migraine in the workplace can be challenging. What are the rights of persons with migraine in the workplace? Time off may provide a brief respite, but it is not a long-term solution. Claiming disability for migraine has challenges, including barriers to doing so and required paperwork. How can persons with migraine remain employed and productive? How can persons with migraine receive accommodation from their employer or take time off to treat their condition?

**Recent Findings:**

Many employers offer comprehensive benefit packages that allow for sick days, time off, mental health days, and accommodations that may give persons with migraine temporary reprieve. However, it may not be sufficient, particularly for those with chronic migraine. Further, not all employers offer comprehensive benefits. Employees with migraine may need to seek protection under the law. Our research closely examines the primary legal avenues that persons with migraine may pursue while living and working with migraine. In exploring the legal aspects of migraine, we couple our exploration with relevant case law.

**Summary:**

Through this lens, we conclude that migraine is becoming less stigmatized and more legitimized in the eyes of the court. It is the belief of the authors that at least some of the change in the attitude towards migraine is the result of strong patient advocacy and significant advances in research over the past several years.

## Summary

This study explores legal aspects of migraine in the workplace. Through relevant case law, we show that courts are starting to recognize migraine as a legitimate medical condition worthy of the same legal protections as conditions with readily observable symptoms.

### Migraine: The Invisible Disease

Migraine is a genetic neurologic disease that affects approximately forty-eight million people in the United States [1]. Migraine is associated with other medical and mental comorbidities, including cardiovascular disease, psychiatric disorders, and sleep disorders [1]. Chronic migraine is associated with higher rates of disability, increased use of healthcare resources, higher direct and indirect costs, lower socioeconomic status, and decreased health-related quality of life [1, 2••].

The effects of migraine in the workplace are staggering. Twenty percent of people with chronic migraine consider themselves “occupationally disabled” [1]. People living with migraine lose 14% of annual productivity [3]. Migraine reduces overall work productivity by 53% [3].

Migraine is not easy to diagnose. Often migraine presents without visible physical symptoms. It can be difficult to show how a person is impacted by migraine. When a person says she or he has a migraine, the diagnosis is based on a belief that this person is telling the truth. Because it is not easy to prove, a person with migraine may suffer twice, from the migraine and from the stigma.

Because migraine is an invisible disease, it raises legal questions for those who experience migraine in the workplace. While the effects of migraine are debilitating, labeling migraine a disability is not cut-and-dried. Classification depends on the individual, the provider, the facts, and the law.

Experiencing migraine in the workplace may result in significant loss of productivity. Presenteeism—physically present but underperforming—is a primary concern for persons with migraine and their employers [4]. How can we help persons with migraine remain productive in the workplace and progressing in their careers without falling into the cycle of presenteeism? And if employees with migraine cannot make it to work, physically or virtually, how can we help them avoid absenteeism?

Many large employers offer comprehensive benefit packages that may give persons with migraine temporary relief from work to treat their condition. However, not all employers offer such benefits. And, for some persons with migraine, such benefits may not be enough. Persons with migraine may need legal protections under federal, state, and local laws to help them remain productive in the workplace.

The good news is that, while historically courts, administrators, and regulatory bodies were hesitant to recognize illnesses, diseases, and conditions that did not have outward, objective manifestations of injury, we are starting to see change. An invisible disease such as migraine is becoming more accepted as a condition worthy of the same legal protections as conditions with visible symptoms and injuries.

## Paid Sick Leave

For persons living and working with migraine, paid sick leave is often the first form of respite. While the Federal Employee Paid Leave Act provides paid sick leave for federal government employees, there is no such mandate for private employers. Because virtually every working person will need time off to care for their own health or that of family members, many state governments have paid sick leave laws.

California, for example, passed the Healthy Workplace Healthy Family Act in 2014 [5]. Under this law an employee who works for 30 or more days within a year from the start of employment is entitled to paid sick leave, accrued at a rate of no less than one hour for every 30 hours worked. An employee can use accrued sick leave beginning on the 90th day of employment. Employers cannot discriminate or retaliate against an employee who takes paid sick leave, but employers can limit an employee’s use of paid sick leave to 3 days per calendar year.

While the California law is silent about proof of illness, for persons with migraine having a doctor’s note is advisable to establish their condition with their employer and, if needed, for a subsequent request for reasonable accommodation and other relief under the various laws discussed herein.

## Employer-Provided Disability Insurance

Term disability insurance provides income replacement for non-job-related injuries or illnesses that render a person unable to work. There are short-term and long-term disability insurance plans. Currently, five states require employees to receive at least short-term disability coverage: California, Hawaii, New Jersey, New York, and Rhode Island. Because employers receive a federal tax deduction for providing short-term disability coverage, most employers offer it. Many employers also offer long-term disability as part of a comprehensive benefits package.

Short-term disability insurance is typically offered for a 90-day period but can range from 30 days up to 1 year. Short-term disability policies have a waiting period which is a defined number of days that a person must be out of work before receiving benefits under the policy. This is known as the “elimination period” and is typically 14 days but can range from 7 to 30 days.

Long-term disability insurance usually starts after the short-term disability policy is exhausted. There may be a waiting period of period of 3 to 26 weeks depending on the short-term policy. A long-term disability policy typically covers 2, 5, or 10 years, or until the insured reaches the age of 65 or 67. The plan may require a person to sign-up for Social Security Disability Insurance (SSDI).

There are several issues persons with migraine may encounter when attempting to use private, employer-provided disability insurance for migraine relief. First, plans will require that such persons exhaust all paid sick leave and other paid time off (PTO) before going out on disability. Second, the disability cannot be job-related. If migraine is job-related, it may be covered by workers’ compensation.

The third issue is that the migraine episodes an employee experiences must qualify as a disability. In context of private disability insurance, there is no standard definition for disability. It varies by state and by insurance plan. In general, a disability is a condition, injury, or illness that renders a person unable to do his or her job. Persons with migraine will need to work with their healthcare provider to document their condition and submit proof to the insurance company that they meet the criteria for disability. It is important to document any absences due to migraine that occurred during the elimination period.

If a disability claim is wrongfully denied by an employer-sponsored plan, the Employee Retirement Income Security Act (ERISA) allows for a private cause of action against the benefits provider [6]. In Valdez v. AT&T, a 2019 case from California, the plaintiff brought such an action against the disability insurance provider for wrongfully denying the plaintiff disability status due to migraine. The court disagreed with the defendant’s contention that migraine is “too subjective” and held that the claim administrator unreasonably discounted the plaintiff’s subjective reports of pain [7••].

## Americans with Disabilities Act

The Americans with Disabilities Act (ADA) is a broad federal law that aims to ensure that people living with disabilities have equal opportunity for employment, among other things [8]. The ADA applies to public and private employers with fifteen or more employees. An employer subject to the ADA cannot fire or refuse to hire a person because of a disability unless an exception applies [9•].

Under the ADA, the person must be a qualified individual with a disability. A qualified individual is a person who can perform the essential duties of the job with or without accommodation. Under the ADA, a disability is defined as a mental or physical impairment that substantially limits one or more major life activities, such as the ability to work or care for oneself.

Under the ADA, an employer must provide reasonable accommodations to an employee with a disability unless doing so would cause undue hardship. An employee with migraine may need reasonable accommodations to prevent the onset of migraine because the workplace often has triggers, including light, sound, and smell. Reasonable accommodations for employees with migraine may include the following:

- Alternative lighting
- Anti-glare filters for fluorescent lights
- Noise cancelling headphones
- Digital screen filters
- Anti-fragrance policies
- Flexible schedules
- Ability to work from home

Under the ADA, the employee must request reasonable accommodation. In addition, supporting documentation from the healthcare provider is typically required by the employer. Employers will approve most reasonable accommodation requests. What is not a reasonable accommodation request under the ADA? There is no bright line, but in Woolf v. Strada—a 2020 case decided by the U.S. Court of Appeals for the Second Circuit—the court held that an employee with migraine requesting a new boss is not reasonable accommodation [10••].

If an accommodation request is denied, the employee should ask for the reason. If the request was reasonable and does not cause the employer undue hardship, the employee can appeal. If unsuccessful on appeal, the employee can file a complaint with the Equal Opportunity Employment Commission (EEOC) or with their state enforcement agency. If unsuccessful with the EEOC, the employee may be able to seek redress through a private lawsuit.

Is migraine a disability under the ADA? It depends. ADA disability is determined on a case-by-case basis rather than a list of conditions. Not everyone with migraine has a disability under the ADA. In the Second Circuit case cited above, the court denied the plaintiff’s migraine disability claim, holding that a person’s inability to perform a singular job or function does not constitute a substantial limitation in the major life activity of working. For such a claim to lie, the plaintiff must show that migraine precludes him from working in a class or broad range of jobs [10••].

## Family and Medical Leave Act

The Family and Medical Leave Act (FMLA) is a federal law that allows eligible employees of a covered employer to take job-protected, unpaid, or accrued paid leave, for up to a total of 12 work-weeks in any 12 months because the employee's own serious health condition makes the employee unable to perform his or her job duties, or because the employee is needed to care for a family member (child, spouse, or parent) with a serious health condition [11].

Does migraine qualify as a serious health condition entitling an employee to FMLA leave? Under the FMLA a serious health condition is defined as an “illness, injury, impairment or physical or mental condition that involves either inpatient care or continuing treatment by a health care provider” [12]. The Code of Federal Regulations states that “headaches other than migraine” do not meet the definition and do not qualify for FMLA leave [13•]. It appears migraine is a serious health condition under the FMLA. However, because the law is not explicit, we look to relevant case law for clarification.

In Alexander v. Boeing Co., the plaintiff worked at Boeing for seventeen years [14••]. During much of her employment plaintiff experienced chronic migraine which management was aware of and had granted accommodations for, including working from home and partial days off. In 2012, plaintiff’s migraine attacks became more frequent, and Boeing stopped allowing partial days off. Plaintiff missed more work, her performance reviews declined, and she was disciplined. In 2013, claiming FMLA leave, plaintiff took 4 days off from work due to a migraine attack. Boeing fired her for “job abandonment.” Plaintiff sued Boeing for FMLA interreference.

In its motion to dismiss the suit, Boeing did not challenge the fact that plaintiff’s migraine constituted a serious health condition, likely because Boeing had documented knowledge of plaintiff’s migraine condition and had accommodated it for years. The takeaway is that under the FMLA, migraine is *presumed* to be a serious health condition. It is incumbent on the employer to rebut or challenge the presumption if it disagrees. However, because the FMLA does not explicitly state that migraine is a serious health condition, ultimately the determination depends on the facts of the case at hand.

Boeing did challenge the sufficiency of notice plaintiff provided when she took leave under the FMLA [15•]. Boeing argued that it required advanced notice. The court disagreed and found that Boeing had not presented any evidence that “plaintiff’s migraines [sic] were foreseeable or otherwise predictable” and the plaintiff was only required to provide notice “as soon as practicable” under the law [16].

## Social Security Disability

Title II of the Social Security Act authorizes disability benefits. The Social Security Disability Insurance (SSDI) program provides financial assistance to qualified individuals with disabilities and their family members. To receive SSDI benefits, the person must have paid sufficient Social Security taxes on their earned income. The Supplemental Security Income (SSI) program pays benefits to those with disabilities who have limited income. Under both programs, monthly benefits are paid if the insured has an approved medical condition that is expected to last at least one year or result in death.

To qualify for SSDI/SSI, a person must have a medically determinable impairment (MDI). Historically, the Social Security Administration (SSA) has been reluctant to recognize as MDIs long-term conditions that do not have physical “signs,” such as migraine. By comparison, the SSA has allowed only 17% of headache disability claims versus 51% for multiple sclerosis (MS) and 78% for Parkinson’s disease [17••].

Recognizing that mobility aids such as canes or walkers are not the only indication of disability, in August 2019, the SSA issued a non-binding Social Security Ruling (SSR) that provides guidance on how the SSA establishes that a person has an MDI of a primary headache disorder and how the SSA evaluates such disorders in disability claims [18].

The SSR guidance states that although primary headache disorder is not a listed impairment, the SSA may find that a primary headache disorder, alone or in combination with another impairment(s), medically equals a listing in the in the SSA Blue Book for Neurological disorders [19]. According to the SSR guidance, Epilepsy (§11.02) is the most closely analogous listed impairment for an MDI of a primary headache disorder. The net result is that persons with migraine seeking disability status for SSDI/SSI benefits must compare their impairment with epilepsy to determine eligibility.

While the SSR is a step in the right direction for persons with migraine, it comes up short. Issuance of the SSR indicates that the SSA is finally acknowledging that migraine is a condition that may cause an SSDI/SSI disability. However, the SSR is medically inaccurate in its requirement that persons with migraine seeking SSDI/SSI disability status compare their disease with epilepsy.

Recent case law suggests the stigma of migraine may be eroding as respects SSDI/SSI disability status. In Tudor v. Saul, plaintiff sought judicial review of an administrative law judge decision denying her application for SSDI benefits [20••]. Plaintiff had enjoyed a distinguished 20-year career as a computer microchip engineer. In September of 2014, plaintiff developed chronic migraine that caused debilitating headaches and prevented her from working. The administrative law judge denied plaintiff’s claim, finding her impairments to be non-severe, and therefore not disabled [21•]. Notably, the administrative law judge found that plaintiff's migraine illness was an MDI. However, the administrative law judge concluded that plaintiff's migraine attacks were caused by a narcotic dependency resulting from opiate medications prescribed to treat the headaches themselves.

The District Court found fault with the administrative law judge’s reasoning on several grounds. The court stated that the administrative law judge overlooked the medical opinions of the plaintiff’s primary care physician and headache specialist in favor of the medical opinions of the physician appointed by the judge. The court also disapproved of the boilerplate language the administrative law judge used to dismiss plaintiff’s own testimony about the severity of her migraine attacks. The court also found that the administrative law judge had used “circular logic” to theorize that plaintiff's migraine was caused by a narcotic dependency. Lastly, the court strongly disapproved of the administrative law judge’s refusal to acknowledge plaintiff’s own testimony about her condition [20••].

The Tudor case is an example of how some administrators responsible for enforcing federal laws rely on outdated assumptions, biases, and document templates when determining the status of persons with migraine. As this case illustrates, courts of review provide crucial checkpoints for ensuring that facts drive the determination of whether a person living with migraine is legally disabled for SSDI/SSI.

## Workers’ Compensation

Workers' compensation is insurance that provides medical benefits and wage replacement to an employee injured in the workplace during the normal course of business. All states except Texas require workers’ compensation [22]. In California, all employers must purchase workers’ compensation insurance, either from the State Compensation Insurance Fund or from a private insurance company or be legally self-insured. Claims are decided by administrative law judges who act as triers of fact [23].

Under workers’ compensation law, injuries arising out of and in the course and scope of employment are compensable. In cases involving migraine, whether migraine is job-related is often at issue. On the one hand, migraine is a genetic neurological disease, that is, a pre-existing condition. On the other hand, the workplace can be a trigger for migraine. The following case addresses this dichotomy.

In Patton v. Paris Henry County Medical Clinic, the plaintiff filed a claim for workers’ compensation benefits against her employer, alleging that her work at the Medical Clinic as an X-ray technician exposed her to chemical odors that caused her to have migraine attacks. When her workers’ compensation claim was denied, plaintiff sued her employer [24••].

Plaintiff had a history of headaches since she was sixteen years old. The headaches that occurred before she worked at the Medical Clinic were occasional tension headaches that were not triggered by any specific foods or odors and were treated with Tylenol. After starting work at the Medical Clinic, plaintiff began experiencing migraine attacks. As she continued her employment, the frequency of migraine attacks progressed to chronic, and plaintiff became disabled as a result.

The employer argued that plaintiff’s migraine attacks did not arise out of and in the course and scope of her employment with the Medical Clinic. In legal terms, the employer argued there was no *causation* between the plaintiff’s alleged injury and her employment at the Medical Clinic because plaintiff had been experiencing headaches since she was a teenager.

The trial court disagreed and ruled in favor of the plaintiff, finding that her injury was compensable and that she is permanently and totally disabled. On appeal, the Tennessee Supreme Court affirmed the trial court decision. The Supreme Court noted that an employer takes an employee “as is” and assumes responsibility for any work-related injury that aggravates a pre-existing injury or previous condition. The court stated that an injury need not be traceable to a definite moment or triggering event to be compensable; rather, an employee may sustain a compensable gradual injury because of continual exposure to the conditions of employment [24••].

Under California Workers’ Compensation law, it has long been established that a physical injury, such as a heart attack, aggravated by work-related stress is compensable. To reduce fraud, in 1989, California amended its workers’ compensation law to explicitly exclude coverage for psychiatric injury substantially caused by lawful, good faith personnel actions [25].

In County of San Bernardino v. Workers’ Compensation Appeals Board, this exception arose in context of a claim for workers’ compensation due to migraine. An employee had been reprimanded for poor job performance. The employee experienced migraine and filed a workers’ compensation claim, which was denied. On appeal, the employee argued that the denial was improper because his work-induced migraine was not a psychiatric injury and thus compensable. While the court agreed that migraine is not a psychiatric injury, the court held that California law precludes recovery for a physiological condition (migraine) that results from psychological injury (stress) caused by good faith personnel action [26••].

## Protecting the Legal Rights of Persons with Migraine

Because the symptoms of migraine are invisible and migraine as a disease may be difficult to prove, it is crucial that healthcare providers help persons with migraine document, thoroughly and accurately, all aspects of their condition.

Persons with migraine should maintain records of all interactions with their employer. If an employee takes paid sick leave due to migraine, the person should notify its employer of the reason. If possible, the employee should provide a doctor’s note even if not required to do so. The purpose here is to put the employer on notice that the employee is living with migraine. Persons with migraine must also have clear documentation of how many hours and days of work they miss due to migraine, and any oral or virtual communications with their employer regarding their migraine condition.

Persons with migraine should maintain a headache journal or diary that includes important details about their attacks, including frequency and intensity, other symptoms such as dizziness, cognitive changes, auras, etc., and how migraine impacts their daily life and livelihood. A well-kept headache diary serves two purposes: medically, it can help the clinician create a comprehensive treatment plan; legally, it can help support a claim of leave or disability due to migraine under one of several laws.

Primary care providers and headache specialists must also maintain spotless records when it comes to treating persons with migraine. Important details include impressions, diagnosis, treatments, and medications. Medical records must be complete, accurate, and current. Providers should have detailed notes for each visit and follow ups, including details about the frequency and intensity of migraine attacks, results of any tests given to rule out other conditions, medications and treatments tried, including outcomes and side effects, and records of any ER visits or hospitalizations related to migraine. Providers should conduct and record the results of standardized functional scales, such as the Migraine Disability Assessment Test (MIDAS) and Headache Impact Test (HIT), both of which can be submitted with paperwork in support of an application for disability or time off due to migraine.

## Conclusion

The impact of migraine on working life can be reduced when persons living with migraine are supported by their employer. If an employee with migraine needs time off from work to treat their condition, short-term or long-term, there are laws in place that the employee can invoke. Although migraine is an invisible disease that has in the past not been accepted by some as a legitimate medical condition, courts are starting to recognize migraine as the debilitating disease it is. For employees living with migraine, it is crucial that they partner with their healthcare provider to document their condition and inform their employer. Programs providing education for employers and advocating for persons with migraine on the individual and policy level have promise to move the needle in the right direction, allowing persons with migraine to live the life they aspire to.
